# Pancreatic Serous Cystadenoma with Compression of the Main Pancreatic Duct: An Unusual Entity

**DOI:** 10.1155/2011/574378

**Published:** 2011-03-14

**Authors:** Stéphanie Truant, D. Izgarevic, Vincent Maunoury, David Buob, Philippe Bulois, Olivier Ernst, Guillemette Huet, Philippe Zerbib, François-René Pruvot

**Affiliations:** ^1^Department of Digestive Surgery and Transplantation, CHU, University of Lille-Nord de France, 59000 Lille, France; ^2^Department of Hepatogastroenterogy, CHU, University of Lille-Nord de France, 59000 Lille, France; ^3^Department of Pathology, CHU, University of Lille-Nord de France, 59000 Lille, France; ^4^Department of Radiology, CHU, University of Lille-Nord de France, 59000 Lille, France; ^5^INSERM U837, Lille 2 University, Jean-Pierre Aubert Centre, University of Lille-Nord de France, 59000 Lille, France

## Abstract

Serous cystadenoma is a common benign neoplasm that can be managed without surgery in asymptomatic patients provided that the diagnosis is certain. We describe a patient, whose pancreatic cyst exhibited a radiological appearance distinct from that of typical serous cystadenoma, resulting in diagnostic difficulties. CT and MRI showed a 10 cm-polycystic tumor with upstream dilatation of the main pancreatic duct (MPD), suggestive of intraductal papillary mucinous tumor (IPMT). Ultrasonographic aspect and EUS-guided fine-needle aspiration gave arguments for serous cystadenoma. ERCP showed a communication between cysts and the dilated MPD, compatible with IPMT. The patient underwent left pancreatectomy with splenectomy. Pathological examination concluded in a serous cystadenoma, with only a ductal obstruction causing proximal dilatation.

## 1. Introduction

Typical macroscopic characteristics of serous cystadenoma of the pancreas consist of microcystic mass with a sponge-like honeycomb aspect or central scar or both. The risk of malignant transformation seems to be low even in the long-term course [1]. Therefore, most authors advise that patients who are asymptomatic can be closely followed provided that differential diagnosis can be definitively made with other potentially malignant cystic tumors, mainly mucinous cystadenoma or intraductal papillary mucinous tumor (IPMT) [2, 3]. But diagnostic difficulties may be encountered in the macrocystic oligocystic form of serous cystadenoma seen in 10% to 30% of cases [4, 5]. In this paper, we present the rare case of a serous cystadenoma of the pancreas causing obstruction and upstream dilatation of the main pancreatic duct, making difficult the differential diagnosis with IPMT.

## 2. Case Report

**Figure 1:**
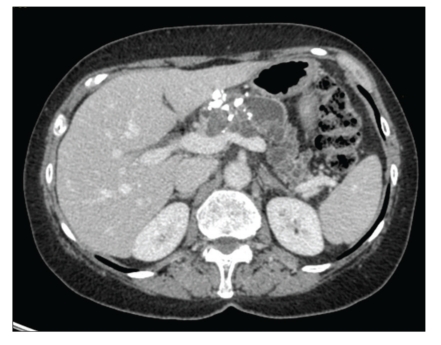
Preoperative computed tomography: presence of a well-defined multiloculated cystic mass in the body and tail of the pancreas with central calcifications.

**Figure 2:**
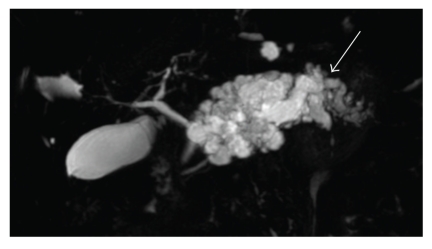
Magnetic resonance cholangiopancreatography: polycystic mass of the left pancreas with upstream dilatation of the main pancreatic duct (arrow). The pancreatic head shows no abnormality and no dilatation of the MPD.

**Figure 3:**
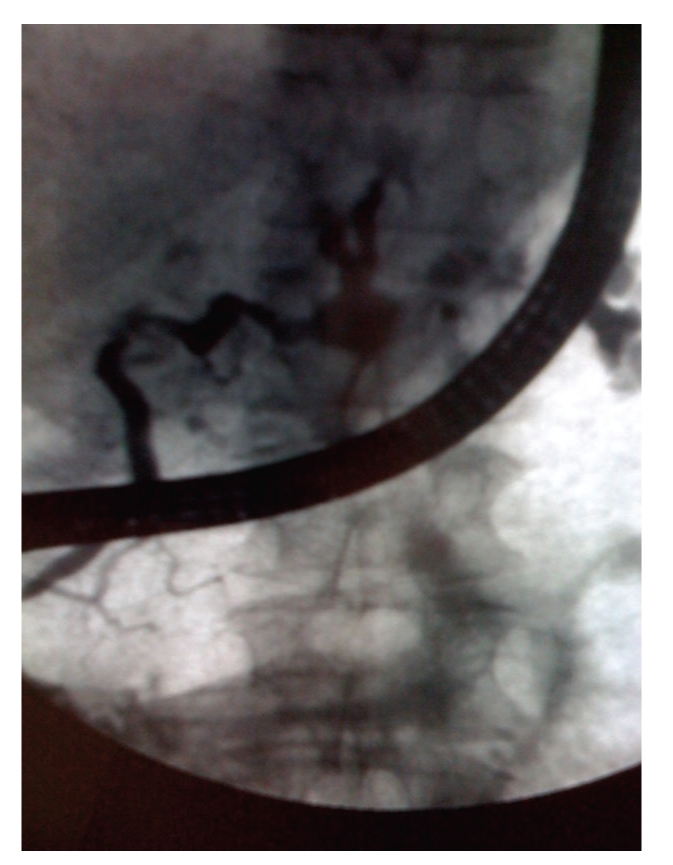
Endoscopic retrograde cholangiopancreatography: cystic dilatation of the pancreatic branches communicating with the dilated MPD suggestive of an IPMT.

**Figure 4:**
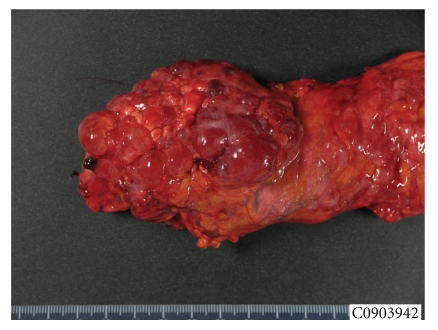
Macroscopic appearance of the pancreatic cystic mass. The lesion, which measures 10 cm in greatest dimension, is made of a combination of large cysts with several small cysts and replaces the entire body and tail of the pancreas.

**Figure 5:**
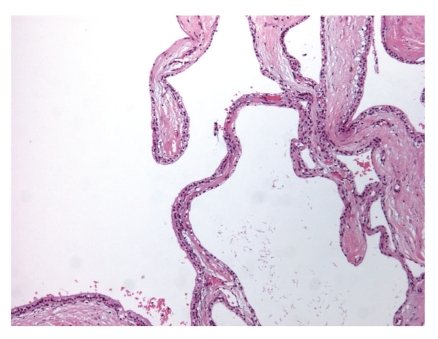
Microscopic appearance of the pancreatic cystic tumor. Low-power microscopic view shows the simple cuboidal epithelial cells with clear cytoplasm.

In January 2009, a 66-year-old woman with a medical history of thyroidectomy was admitted to Lille University Hospital for further evaluation of a large cystic mass in the distal pancreas. She had complained of transient epigastric pain one month before. Blood biochemical parameters showed no pancreatitis and the tumor markers, mainly CEA and CA 19-9, were all within the normal limits. On admission, the patient was asymptomatic and the pancreatic mass was not palpable in the upper abdomen. Contrast-enhanced computed tomography (CT) and magnetic resonance imaging (MRI) showed a huge, well-defined, multiloculated, cystic mass, of 10 cm in greatest dimension, replacing the entire body and tail of the pancreas, and displaying multiple calcifications in its isthmic part (Figures 1 and 2). MRI also showed an upstream dilatation of the main pancreatic duct (MPD) to 16 mm, with no dilatation of its distal part and no abnormality in the pancreatic head (Figure 2). Both MRI and CT concluded in IPMT. By contrast, endoscopic ultrasonography (EUS) showed a component of microcystic pattern and concluded in a serous cystadenoma. EUS-guided fine-needle aspiration (FNA) was performed through the gastric wall in order to analyze the cyst fluid at biochemical and cytological levels: concentration of CEA was 0.2 ng/mL, while cytological analysis was not contributory. Regarding the diagnostic uncertainty, we performed an endoscopic retrograde cholangiopancreatography (ERCP) that showed a cystic dilatation of the pancreatic branches communicating with the dilated MPD suggestive of an IPMT (Figure 3). These results prompted us to operate on the patient especially as she had complained of abdominal pain. On exploratory laparotomy, a huge multicystic tumour measuring 10 cm in maximum diameter was found, replacing the entire body and tail of the pancreas and surrounding the splenic vessels. A distal pancreatectomy combined with splenectomy was carried out. The postoperative course was uneventful, and the patient was discharged on day 9. Macroscopic examination of the resected specimen showed a combination of large cysts with several small cysts and central calcifications (Figure 4). There was no communication between the ducts and the cysts, but a compression of the main pancreatic duct and secondary branches by the cysts with upstream dilatation. Microscopically, the cysts were lined by a single layer of cuboidal epithelial cells with clear cytoplasm (Figure 5). Histopathological examination was thus indicative of serous cystadenoma of the pancreas. Six months after surgery, the patient remains well and asymptomatic.

## 3. Discussion

Serous cystadenoma has 2 main morphologic patterns: typical one, seen in 70% of cases, displays microcystic pattern that is characterized by radiologically visible multiple cysts measuring 2 cm or smaller, without communication with the MPD [5–7]. The cystic spaces are separated by fibrous septa that can coalesce into a central scar that may calcify [8]. Delayed imaging may occasionally be helpful for depiction of the central scar [9]. In approximately 20% of patients, serous cystadenomas are characterized by a honeycomb pattern with numerous subcentimeter cysts which are not radiologically distinguishable. 

However, there are various features of serous cystadenomas found on imaging studies which may lead to diagnostic difficulties. Main atypical manifestations include cystic tumor without microcystic pattern, cystadenoma with interval growth, cystadenoma communicating with the MPD, and giant tumors with ductal dilatation as seen in our patient [5, 7]. Serous macrocystic adenoma that is composed of only a few relatively large cysts (>2 cm) or even a unilocular cyst comprised 10% to 30% of the serous cystadenomas [10]. Several cases of serous oligocystic cystadenoma have been misdiagnosed as mucinous cystadenoma and inappropriately managed [11, 12]. Serous cystadenoma may display interval growth, with larger tumors (≥4 cm at presentation) more likely to grow faster than small ones, up to 2 cm per year [7]. Communication with the MPD is not a usually finding with serous cystadenomas and was found at ERCP in only 0.6% of cases in a series of 144 serous cystadenomas [13–15]. Giant serous cystadenomas are also rare; this term usually refers to a multicystic tumor larger than 10 cm in diameter in comparison with a mean tumor diameter of 4 to 5 cm in most reports of serous cystadenoma [1, 16, 17]. In some rare cases, the giant lesions produce a symptomatology caused by the compression of the MPD, as seen in our patient, or of adjacent structures such as bile ducts or colon [1, 18]. Serous cystadenoma with ductal dilatation is a rare cause of obstructive acute or chronic pancreatitis [19]. When dilatation is observed, differential diagnosis with IPMT may be difficult. Interestingly, Kim et al. [2] have shown in 41 patients that diffuse or distal MPD dilatation was exclusively observed in IPMT, whereas proximal MPD dilatation tended to be observed in serous cystadenoma. Diffuse MPD dilatation in IPMT was mostly associated with mucin secreted from the tumor, whereas proximal MPD dilatation in serous cystadenoma was probably a mass effect due to extrinsic compression [2].

In most cases, the diagnosis of cystic pancreatic lesions relies on CT and MRI. Nevertheless, when combined, their diagnostic accuracy was less than 50% of cases in a recent series of 70 cystic pancreatic lesions independently reviewed by two blinded radiologists [13]. ERCP proves especially useful in patients with IPMT, demonstrating mucin at the ampulla and diffuse dilatation of the pancreatic ducts. Nevertheless, in our case of compressive serous cystadenoma, ERCP by showing a subsequent dilatation of the main pancreatic duct and secondary branches perplexed us. EUS has been proposed as an ideal imaging technique for pancreatic cystic lesions, as it offers two means of diagnosis, that is, high-resolution morphologic imaging and guidance for FNA [20]. While EUS imaging alone has limitations regarding definitive diagnosis, aspiration, and characterization of cyst fluid contents, that is, cytology, mucin, and tumor markers, may provide incremental information. Although cytology alone is rarely definitive, when such cytologic samples are positive, the specificity is high [7]. Regarding cyst fluid tumor markers, the Cooperative Pancreatic Cyst Study [21] reported that a CEA level greater than 192 ng/mL had a sensitivity, specificity, and diagnostic accuracy of 73%, 84%, and 79%, respectively, for mucinous cystic lesions. A later study identified cut-off levels of 5 ng/mL to be highly specific (95%) for nonmucinous cysts, as seen in our patient [22]. Consistently, cyst fluid CEA of less than 5 ng/mL for the diagnosis of nonmucinous lesions had a sensitivity of 44%, specificity of 96%, and diagnostic accuracy of 78% in a recent study [23]. Although there are differences in CEA levels by cyst type, there is also substantial overlap, and this is particularly true for other cyst fluid tumor markers [24, 25]. False-negative results, in which there is no elevation of CEA, are also possible but rarely seen [26]. Although not performed in our centre, assessment of cyst mucin may provide additional information, with the best profile obtained when both mucin and CEA are determined along with cytology in a recent study [26]. 

**Figure 6:**
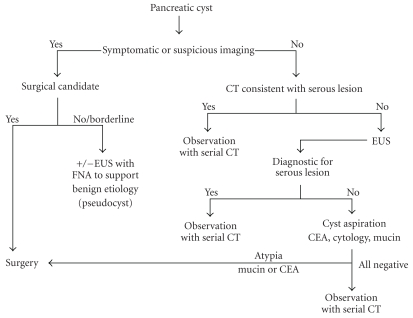
Diagnostic and management algorithm for cystic lesion of the pancreas.

Patients bearing a cystic pancreatic lesion are advised to undergo resection based on defined criteria: the presence of symptoms, abnormal cyst aspiration fluid, and radiologic criteria suspicious for a mucinous neoplasm (main duct and mixed type IPMN, cysts with associated mass, haemorrhage or mural nodule, duct obstruction, or cyst rim calcifications) [27]. By contrast, for asymptomatic patients with benign-appearing lesions, such as classic appearance of a serous cystadenoma, observation alone seems appropriate [28]. Some authors have nevertheless advised resection for serous cystadenomas measuring 4 cm or more in maximal diameter regardless of the presence or absence of symptoms, because of greater median growth rate [7]. Regarding asymptomatic lesions with uncertain diagnostic, it is important to identify those lesions in need of resection and those that may be safely monitored. It is in this cohort that FNA may be most beneficial. In those patients, EUS with FNA confirmation of a negative cytology and low fluid CEA can further provide evidence to support a monitoring approach and deferral of surgical intervention [28]. In such a case where preoperative distinction between low-risk pancreatic cysts such as serous cystadenoma from high-risk neoplastic mucinous cysts (mucinous cystadenoma and IPMT) cannot be made with absolute certainty, laparotomy is inevitable, even for serous cystadenomas incidentally discovered [16, 17]. Our algorithm for diagnostic and management of pancreatic cystic lesions is summarized in the Figure 6.

## 4. Conclusion

In conclusion, our case highlights an extremely rare case of serous cystadenoma with compression of the main pancreatic duct. Despite the availability of high-quality imaging techniques, uncertain diagnosis led us to perform left pancreatectomy, although EUS with FNA were suggestive of serous cystadenoma. Algorithm is then proposed to manage cystic pancreatic lesions: (i) symptomatic lesions must be removed and (ii) EUS with FNA is the key investigation for asymptomatic lesions of uncertain imaging diagnosis.
